# The association of very low-density lipoprotein receptor (VLDLR) haplotypes with egg production indicates VLDLR is a candidate gene for modulating egg production

**DOI:** 10.1590/1678-4685-GMB-2015-0206

**Published:** 2016-07-14

**Authors:** ZhePeng Wang, GuoHua Meng, Na Li, MingFen Yu, XiaoWei Liang, YuNa Min, FuZhu Liu, YuPeng Gao

**Affiliations:** College of Animal Science and Technology, Northwest A&F University, Yangling, Shaanxi, China

**Keywords:** chicken, egg production, haplotype, very low-density lipoprotein receptor (VLDLR)

## Abstract

The very low-density lipoprotein receptor (VLDLR) transports egg yolk precursors into oocytes. However, our knowledge of the distribution patterns of VLDLR variants among breeds and their relationship to egg production is still incomplete. In this study, eight single nucleotide polymorphisms (SNPs) that account for 87% of all VLDLR variants were genotyped in Nick Chick (NC, n=91), Lohmann Brown (LohB, n=50) and Lueyang (LY, n=381) chickens, the latter being an Chinese indigenous breed. Egg production by NC and LY chickens was recorded from 17 to 50 weeks. Only four similar haplotypes were found in NC and LohB, of which two accounted for 100% of all NC haplotypes and 92.5% of LohB haplotypes. In contrast, there was considerable haplotypic diversity in LY. Comparison of egg production in LY showed that hens with NC-like haplotypes had a significantly higher production (p < 0.05) than those without the haplotypes. However, VLDLR expression was not significantly different between the haplotypes. These findings indicate a divergence in the distribution of VLDLR haplotypes between selected and non-selected breeds and suggest that the near fixation of VLDLR variants in NC and LohB is compatible with signature of selection. These data also support VLDLR as a candidate gene for modulating egg production.

## Introduction

In the poultry industry, egg production is an important trait that determines economic profits. Therefore, understanding the genetic factors that govern this trait is of great significance to geneticists, breeders and producers. However, because of the complex genetic factors and possible environmental influences that can interfere with egg production, our knowledge of the molecular regulatory mechanisms underlying this trait is still limited.

Egg production involves the development of oocytes and ovulation. In this process, the oocytes will take up precursors for yolk formation from the circulation, grow from 6-7 mm to 35 mm and finally undergo ovulation (Bujo *et al.*, 1997). Very low-density lipoprotein (VLDL) and vitellogenin (VTG), two key yolk precursors that transport lipids and account for more than 30% of yolk weight, are taken up by oocytes via receptor-mediated endocytosis (Bujo *et al.*, 1997).

The very low-density lipoprotein receptor (VLDLR) is the key carrier of VLDL and VTG (Bujo *et al.*, 1994), which suggests that changes in the expression and function of the VLDLR gene may influence oocyte development and subsequent egg production. This inference is supported by a study showing that hens with a non-synonymous mutation involving C682S (defined as the RO mutation) in the VLDLR fail to lay eggs and display sever hyperlipidemia (Bujo *et al.*, 1995). The level of ovarian VLDLR expression is also correlated with the rejuvenation of reproductive performance in molted hens (Meng *et al.*, 2013) and with egg mass, clutch size and laying interval in zebra finch (Han *et al.*, 2009).

With regard to the mapping of quantitative trait loci (QTL), several genome-wide association and selective sweep analyses have attempted to identify genomic regions associated with egg production and quality traits by using high-density single nucleotide polymorphism (SNP) chips or whole-genome sequencing (Rubin *et al.*, 2010; Liu *et al.*, 2011; Elferink *et al.*, 2012; Wolc *et al.*, 2012, 2014; Gholami *et al.*, 2015; Sun *et al.*, 2015a, b; Yi *et al.*, 2015; Yuan *et al.*, 2015). However, because of lower genetic variation on chromosome Z (Sundström *et al.*, 2004) and the limitations of statistical methods, the Z chromosome has been consistently excluded from most studies (Rubin *et al.*, 2010; Elferink *et al.*, 2012; Gholami *et al.*, 2015; Sun *et al.*, 2015a, b; Yi *et al.*, 2015; Yuan *et al.*, 2015), or limited Z-linked SNPs have been used (Liu *et al.*, 2011; Wolc *et al.*, 2012, 2014). This means that the Z-linked VLDLR, despite being a functionally important candidate, has not been identified by high-throughput mapping studies. Nevertheless, the results of some low-accuracy mappings have suggested that the VLDLR is a promising candidate gene for modulating egg production. Specifically, several VLDLR variants show a significant association with egg weight, age at first egg and egg production in chickens, ducks and quails (Wang *et al.*, 2011; Cao *et al.*, 2012; Wu *et al.*, 2015). QTL intervals for egg number and age at first egg cover the VLDLR (Tuiskula-Haavisto *et al.*, 2002; Sasaki *et al.*, 2004; Xu *et al.*, 2011) and the mapping intervals for Haugh units, egg weight, yolk height and egg production rate locate near the VLDLR (Atzmon *et al.*, 2007; Honkatukia *et al.*, 2013; Goto *et al.*, 2014).

Despite the foregoing studies, there is still only limited information on the distribution patterns of VLDLR variants among chicken breeds and the relationship between these variants and egg production. The aim of this work was therefore to examine the sequence variants of VLDLR among Nick Chick (NC), Lohmann Brown (LohB) and Lueyang (LY) chickens, the latter being a Chinese indigenous breed. The relationship between VLDLR haplotypes, VLDLR mRNA expression and egg production was also analyzed.

## Material and Methods

### Birds and data collection

Three breeds of chickens (NC, LohB and LY) were used in this study. NC and LohB represent commercial white- and brown-egg layer breeds, respectively, specifically selected for a spectrum of egg production and quality traits. LY is a domesticated unselected indigenous breed from Lueyang town in Shaanxi province, China. Historically, LY chickens have not been specifically selected for production traits. Consequently, the breed shows very poor egg production performance and large intraspecies variability, with an average of 85-115 eggs per year.

Fertilized NC eggs were collected from 135 half-sib families whereas fertilized LY eggs were from a free-range, random-mating population of approximately 1,800 birds with male:female ratio of 1:20. Two experimental populations of 500 NC (450 hens and 50 roosters) and 2,853 LY (1,543 hens and 1,310 roosters) born in the same hatch were reared on the experimental station at Northwest A&F University from December, 2012 to November, 2013. These birds were reared in single-hen cages with feed and water *ad libitum*. Egg production of 100 NC and 500 LY that were randomly selected from the above experimental populations was recorded from 17 to 50 weeks of age. Finally, 91 NC and 381 LY with complete egg production records were used in the subsequent association study. Fifty LohB were randomly sampled from a commercial population of approximate 20,000 birds.

### Estimation of egg production parameters

The weekly number of eggs per bird was recorded from 17 to 50 weeks of age and the individual egg production rate was calculated as the weekly number of egg divided by seven days. Based on the data, several mean population egg production parameters were estimated using the three nonlinear regression models indicated below:

1. Segmented polynomial model (Fialho and Ledur, 1997):

$$y=\left\{ \begin{array}{l} p-3\times p\times {\left(\frac{tp-t}{tip}\right)}^{2}+2\times p\times {\left(\frac{tp-t}{tip}\right)}^{3},tp-tip\leq t\leq tp\\ \;p-s(t-tp),\;tp\leq t, \end{array}\right.$$

where **p** is the peak egg production rate, **s** is the weekly decrease in egg production rate after the peak, **tp** is the age of the hen at the peak and **tip** is the time interval between the start and peak of egg production.

2. Yang model (Yang *et al.*, 1989):

$$y=\frac{\text{a}e^{-\text{xt}}}{1+e^{-\text{c}(\text{t}-\text{d})}}$$

where **a** is a scale parameter, **c** is a reciprocal indicator of the variation in sexual maturity, **x** is the weekly decrease in egg production rate after the peak production and **d** is the mean age of sexual maturity of the hens.

3. Persistency model (Grossman *et al.*, 2000):

$$\begin{array}{l} y=0.3\times \left(\frac{yp}{t2-t1}\right)\times \left[\ln\left(\frac{e^{\frac{t}{0.3}}+e^{\frac{t1}{0.3}}}{1+e^{\frac{t1}{0.3}}}\right)-\ln\left(\frac{e^{\frac{t}{0.3}}+e^{\frac{t2}{0.3}}}{1+e^{\frac{t2}{r0.3}}}\right)\right]+\\ 0.3\times b4\times \ln\left(\frac{e^{\frac{t}{0.3}}+e^{\frac{\left(t2+p\right)}{0.3}}}{1+e^{\frac{\left(t2+p\right)}{0.3}}}\right) \end{array}$$

where **yp** is the egg production rate at the peak production, **t1** is the time at the transition from a slow increase to a rapid increase in the egg production rate, **t2** is the time at the transition from a rapid increase to a constant rate of egg production, **P** is the duration of the period of constant production, and **b4** is the weekly decrease in egg production rate after constant production.

All parameters were estimated using the nonlinear least-square method by searching for the set of parameters that produced the smallest sum of the squared errors. The nonlinear least-squares estimate was obtained by using the Gauss-Newton algorithm in the NLIN procedure of SAS v9.2 software with a default convergence criterion of 10^−5^ (SAS Institute Inc., 2008).

### Evaluation of the goodness of fit of the three nonlinear models

Four statistics, namely, Akaike's information criterion (AIC), mean square error (MSE), coefficient of determination (R^2^) and mean model error (MME), were used to evaluate the goodness of fit of the three nonlinear models.

#### AIC

The AIC is a statistic based on information theory. In contrast to R^2^ (see below), AIC cannot evaluate the quality of a model in an absolute sense. Rather, given a set of candidate models, AIC can provide a relative estimate of the quality of these models, with the preferred model being the one with the minimum AIC value. AIC is calculated as follows:

AIC = n × Ln(SS_error_/n) + 2 × k (Savegnago *et al.*, 2012)

where **n** is the number of records used for parameter estimation, **SS**_error_ is the sum of the squared error of the model, and **k** is the number of parameters in the model.

#### MSE

MSE is calculated as follows:

$$\text{MSE}=\frac{\sum_{i=1}^{n}\sum_{t=1}^{m}{\left(y_{it}-{\hat{y}}_{it}\right)}^{2}}{nm-p}$$

where **y**_it_ is the observed weekly egg production rate of hen *i* at week *t*, ${\hat{\text{y}}}_{\text{it}}$ is the predicted weekly egg production rate of hen *i* at week *t*, **n** is the number of hens, **m** is the number of weeks during which egg production was recorded, and **p** is the number of parameters in the model.

#### R^2^

R^2^ is calculated as follows:

$${\text{R}}^{2}=\frac{{\text{SS}}_{\text{model}}}{{\text{SS}}_{\text{total}}}$$

where SS_model_ is the sum of the squares of the model and SS_total_ is the total sum of the squares.

#### MME

MME is the mean of all model errors. One advantage of MME is that the statistic not only evaluates the goodness of fit of a model, but also reflects the direction in which observed values deviate from predicted values (Savegnago *et al.*, 2012). A positive MME means that the model overestimates the egg production rate as a whole; correspondingly, a negative MME indicates that the egg production rate is underestimated. MME is calculated as follows:

$$\text{MME}=\frac{\sum_{t}^{m}\frac{\left({\hat{y}}_{t}-{\bar{y}}_{t}\right)}{{\bar{y}}_{t}}}{m}$$

where ${\hat{y}}_{t}$ is the average predicted egg production rate at week *t*, ${\bar{y}}_{t}$ is the average observed egg production rate at week *t*, and **m** is the number of weeks during which egg production was recorded. In this study, **m** was equal to 34 (from 17 to 50 weeks).

The flexibility of the models and the differences in nonlinear trends among groups were assessed by plotting and analyzing the fitted egg production curves and the average observed weekly egg production rates.

### Comparison of egg production parameters among different groups

Differences in the egg production parameters between LY and NC and among four haplotype groups were compared statistically using the sum of squares reduction test (SSRT; SAS Institute Inc., 2008). The null hypothesis for the SSRT, also known as a reduced model, is that there is no significant difference (p > 0.05) in the nonlinear regression trends between groups such that the same set of parameters should exist across groups. In contrast, the alternative hypothesis, corresponding to a full model, assumes that nonlinear trends should vary across groups and that different sets of parameters are required to fit the egg production rates of different groups. The main idea of SSRT is to statistically indicate whether the full model, which applies more sets of parameters, provides a significantly better fit than the reduced model. An F_R_ statistic was calculated as:

$$\text{FR}=\frac{\left({\text{SSE}}_{\text{r}}-{\text{SSE}}_{\text{f}}\right)/\left({\text{df}}_{\text{r}}-{\text{df}}_{\text{f}}\right)}{{\text{SSE}}_{\text{f}}/{\text{df}}_{\text{f}}}$$

where SSE_r_ and SSE_f_ indicate the residual sum of squares of the reduced model and full model, respectively, with df_r_ and df_f_ being the error degrees of freedom. Because the difference df_r_ – df_f_ corresponds to the number of increased parameters in the full model, the numerator of the F_R_ statistic can be explained as the mean contribution that every additional parameter in the full model makes to the reduction in the residual sum of squares. After scaling the full model by the mean squared error, the contribution is statistically evaluated by comparing the F_R_ statistic and quantiles from an F distribution with the (df_r_ – df_f_) numerator and df_f_ denominator degrees of freedom.

In the event of the null hypothesis being rejected, it is necessary to elucidate whether all parameters, or only some of them, varied across groups. For this, parameter differences were examined using the method mentioned in Example 60.5 of the SAS/STAT^®^ 9.22 User's Guide (SAS Institute Inc., 2008). Briefly, parameter differences (Δpar) between two groups and the 95% confidence interval of Δpar were estimated by a simple reparameterization in which Par2 (parameters of group 2) were replaced by Par1 (parameters of group 1) + Δpar. The statistical significance of Δpar would be confirmed if the 95% confidence interval of Δpar excluded 0 (SAS Institute Inc., 2008). The SSRT was run using SAS 9.2 software (SAS Institute Inc., 2008).

### Analysis of VLDLR genomic sequence variants

Blood was collected from a wing vein into anticoagulant (ACD – acid citrate dextrose solution) and immediately stored at −20 °C. Genomic DNA was extracted from blood using the phenol/chloroform method (Sambrook and Russell, 2006). A genomic interval of chromosome Z (chrZ; 26411455-26431662) covering the whole VLDLR gene and a 5 kb upstream region was re-sequenced using 10 LY chickens randomly selected from the 381 specimens mentioned above. The primers used for polymerase chain reaction (PCR) amplification and Sanger sequencing are listed in Table S1. VLDLR sequence variants were found by sequence alignment using ChromasPro 1.5.

SNP genotypes were detected by PCR-restriction fragment length polymorphism (PCR-RFLP). The primers and restriction enzymes used in PCR-RFLP are shown in Table 1. Part of the PCR-RFLP results were also verified by Sanger sequencing.

**Table 1:** Primer sets and restriction enzymes used to genotype 8 tag SNPs.

Description^a^	dbSNP accession number^b^	Position in VLDLR	Primer sequences	Restriction enzymes
g.26 419 086 G > A	rs312633123	Intron 1	F: 5'-AAAGCCTGGATAAGAGCG-3'	*Hae*III
			R: 5'-TTCCAAATGAAGGGAAGC-3'	
g.26,420,908T > G	novel SNP	Intron 2	F: 5'-TAACAGCAGGAAGTGGAT-3'	*Hin*fI
			R: 5'-GCATACTAATGGCAACAA-3'	
g.26,423,124A > G	novel SNP	Intron 4	F: 5'-TCAGGGTTTCTTAACAGC-3'	*Sna*BI
			R: 5'-ACACCTCAGGCAACTCAT-3'	
g.26,424,657G > A	rs317219970	Exon 6 (synonymous mutation)	F: 5'-GATGAAATCAACTGCCGTAA-3'	*Xba*I
			R: 5'-CAGGTCCAGAACACTGAATAAC-3'	
g.26426398T > C	rs317314828	Intron 10	F: 5'- GCCAAATCTGTATCAACC-3'	*Mfe*I
			R: 5'- ACGGGTATCAATAGAGGC-3'	
g.26,427,922AG	rs314567990	Intron 14	F: 5'-CGTGTATTCTGGATTGACGGAG-3'	*Hinf*I
			R: 5'-CAGCAGGGGTGCTAGGCT-3'	
g.26,429,604A > C	rs14777635	Intron 15	F: 5'-ATGAAATGCCAAGAGTGC-3'	ApoI
			R: 5'-AAGTAGTGAGGCTGCTTA-3'	
g.26,431,186C > T	rs314284862	3' UTR	F: 5'-ACGGGTTACTATGATTGC-3'	*Bse*RI
			R: 5'-TCCAGCCAGACTCTTACA-3'	

a SNP positions on chromosome Z are based on the chicken genome galGal4 assembly.

b Accession number in the dbSNP database (http://www.ncbi.nlm.nih.gov/snp)

### Detection of the RO mutation in LY chickens

Fifty LY hens with an egg number < 100 from 17 to 50 weeks and 30 LY roosters were selected from the 381 LY hens and 1,310 roosters. The RO mutation was identified using PCR-RFLP. A 559 bp fragment containing the RO locus was obtained by PCR using the forward primer 5'-TCTATGGTGCCAACAAAT-3' and the reverse primer 5'-CATCTCAGACCGTCCTCC-3'. After digestion of the PCR products with *Eco*57I (Life Technologies, Shanhai, China) at 37 °C for 2 h, the products were separated on a 2% agarose gel. Since the 559 bp fragment contained two *Eco*57I cleavage sites at 85 bp and 451 bp, wild-type birds should show two bands (85 bp and 474 bp), whereas those with the RO mutation should have four bands (85 bp, 474 bp, 108 bp and 451 bp).

### Detection of VLDLR mRNA expression in ovary and liver

The levels of VLDLR mRNA expression in ovary and liver were compared among NC (n=6) and LY chickens with (n=6) and without (n=6) the ATAATA(A/C)T haplotypes. All birds were 34 weeks old and were selected from the LY and NC populations described above. The birds were rapidly killed after being anaesthetized with 5 mg/kg of Zoletil by intramuscular injection. Liver and ovary with previtellogenic follicles (diameter < 5 mm) were immediately removed and immersed in RNAlater solution (CWBio Corp., Beijing, China) at 4 °C overnight and then stored at −80 °C until used. The use and care of birds in this study was approved by the Northwest A&F University Ethics Committee.

Total RNA was extracted from tissues using TRIzol (CWBio Corp.), according to the manufacturer's instructions. The quality (intactness) of the RNA was assessed visually after electrophoresis in 1% agarose gels and further confirmed by an RNA integrity number (RIN) [#GTEQ#] 7 provided by BioAnalyzer 2100 (Agilent Technologies Corp., Santa Clara, CA, USA). One microgram of total RNA was transcribed to cDNA with a HiFi MMLV first-strand cDNA synthesis kit (CWBio Corp.), according to the manufacturer's instructions. Briefly, RNA was added to 20 μL of reaction mixture that consisted of 4 μL of dNTP mix (2.5 mM each), 2 μL of oligo dT primer (20 μM), 4 μL of 5RT buffer (250 mM Tris-HCl, pH 7.6, 375 mM KCl, 15 mM MgCl_2_), 2 μL of 0.1 M DTT, 1 μL of 200 U of HiFi-MMLV/μL and RNase-free water. The reaction conditions were 42 °C for 45 min for cDNA synthesis and then 85 °C for 5 min to inactivate MMLV reverse transcriptase.

The expression of VLDLR in ovary and liver was detected using quantitative real-time PCR (qPCR). The reactions were run using an UltraSYBR mixture qPCR kit (CWBio Corp.) in a total volume of 20 μL containing 1 μL of cDNA, 0.3 μL of primer pairs (4 μM each), 10 μL of 2 UltraSYBR mixture and 8.4 μL of RNase-free water. The reactions were run in an iQ5 real-time PCR detection platform (Bio-Rad Laboratories, Inc, Hercules, CA, USA) using the cycling conditions described in the UltraSYBR mixture protocol sheet. Three technical replicates were run for every sample. The resulting Ct data were analyzed using the 2^−^ΔΔCT method (Livak and Schmittgen, 2001). GAPDH was used as an internal reference (housekeeping gene) to normalize the amount of cDNA input. Samples from birds without the ATAATA(A/C)T haplotypes were used as calibrators and the level of VLDLR expression in birds with ATAATA(A/C)T haplotypes and in NC chickens was expressed as the fold-change relative to the calibrator. The forward and reverse primers were 5'-TGTGGTCCTCAGTCAACC-3' and 5'-TCTGCTGCACTACAAGTCA-3' for VLDLR and 5'-ATACACAGAGGACCAGGTTG-3' and 5'-AAACTCATTGTCATACCAGG-3' for GAPDH.

## Results

### Description of the goodness of fit of the three nonlinear models

**Figure 1:**
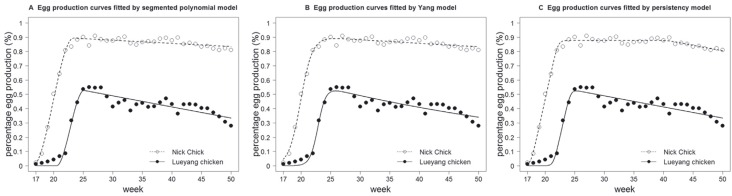
Average weekly egg production rate and fitted egg production curves for Lueyang and Nick Chick chickens.

**Figure 2:**
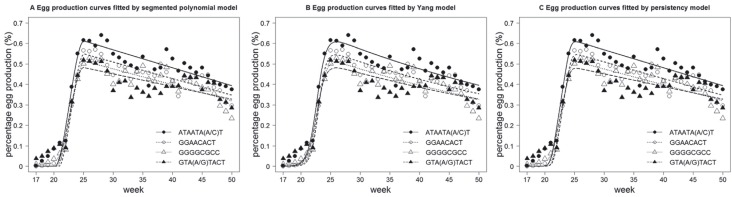
Average weekly egg production rates and the fitted egg production curves for different LY haplotypes. ATAATA(A/C)T represented two haplotypes (ATAATAAT and ATAATACT), as did GTA(A/G)TACT.

Egg production records from the LY, NC and four LY haplotype subgroups were fitted using the Segmented Polynomial, Yang and Persistency models. The quality of fit of the models was assessed statistically (Table 2) and graphically (Figures 1 and 2). The three models showed similar goodness of fit when dealing with the data from the LY or NC, which was supported by the almost identical AIC, MSE and R^2^ values (Table 2). Exceptionally, the MME value showed considerable variation among the three models. For the NC group, the MME statistic suggested that the Segmented Polynomial model provided the best fit, whereas for the LY and four LY haplotype subgroups the MME values were consistently close to zero in the Yang model, which suggested that this was the best model (Table 2). Compared to the weak effect of the models *per se* on the goodness of fit, the data themselves exerted a more important role in determining the goodness of fit. Thus, higher R^2^ and lower MSE and MME indicated that the goodness of fit of the three models was better for NC data than for LY (Table 2).

**Table 2:** Statistical criteria used to evaluate the goodness of fit of three nonlinear models in fitting the data from Lueyang and Nick Chick chickens^a^

Groups	Models	AIC	MSE	R^2^	MME
NC	Segmented polynomial	−6491.4	0.0219	0.968	−0.00124
	Yang	−6493.9	0.0219	0.967	0.0393
	Persistency	−6489.6	0.0219	0.968	−0.0116
LY	Segmented polynomial	−22892.2	0.0748	0.663	−0.119
	Yang	−22906.5	0.0747	0.663	−0.110
	Persistency	−22893.2	0.0748	0.663	−0.127
ATAATA(A/C)T	Segmented polynomial	−2674.7	0.0646	0.758	−0.114
	Yang	−2678.9	0.0644	0.759	−0.0965
	Persistency	−2672.4	0.0647	0.758	−0.116
GGAACACT	Segmented polynomial	−5356.7	0.0735	0.684	−0.104
	Yang	−5356.7	0.0734	0.684	−0.0716
	Persistency	−5354.7	0.0734	0.684	−0.100
GGGGCGCC	Segmented polynomial	−6283.3	0.0747	0.649	−0.115
	Yang	−6285.9	0.0746	0.649	−0.101
	Persistency	−6281.3	0.0746	0.649	−0.123
GTA(A/G)TACT	Segmented polynomial	−4361.6	0.0761	0.629	−0.102
	Yang	−4366.9	0.0759	0.629	−0.0958
	Persistency	−4359.6	0.0761	0.628	−0.101

a AIC – Akaike's information criterion, MSE – mean square error, R^2^ – coefficient of determination, MME – mean model error.

By plotting the fitted curve and the average observed weekly egg production rates, it was possible to graphically assess the flexibility of the models. In line with results from the statistical evaluation, all three models displayed excellent flexibility in adjusting to changes in the egg production rate of NC. However, deviations of the fitted curves from the data for LY were severe, especially for the egg production rates from 17 to 21 weeks, for which these models did not show enough flexibility to slow the increase in egg production rates (Figure 1).

### Comparison of egg production between LY and NC chickens

By using the three nonlinear models, 13 egg production parameters were estimated and used to evaluate egg production performance in LY and NC. These parameters were classified into two categories: category 1 evaluated the changes in egg production rate, such as peak production rate (parameters **P** and **yp**) and the decrease in egg production rate after the peak (**s, x** and **b4**); category 2 evaluated the changes in egg production time, such as age at the first egg ((**tp-tip**), **t2**), time to reach the production peak (**tp** and **t2**), sexual maturity (**d**) and the maintenance of constant production (persistency) (Table 2). LY chickens had a lower production peak (0.53 *vs*.0.89 for **P**), larger decrease in the egg production rate (0.0078 *vs.* 0.0022 for **s**), longer time until the start of egg laying (21.4 *vs.* 17.5 for **t1**), peak production (25.1 *vs.* 23.3 for **tp**) and sexual maturity (22.9 *vs.* 19.9 for **d**), as well as zero persistency (0 *vs.* 18.5), when compared to NC (Table 3, Figure 1). The differences in these parameters indicated that egg production was lower in LY than in NC.

**Table 3:** Comparison of the egg production parameters in Lueyang and Nick Chick chickens.

Egg production parameters	LueYang chicken	Nick Chick	Δpar^a^	95% Confidence interval of Δpar^b^	F value, P value^c^	Model
n	381	91	–			
Record N	8829	3094	–			
P	0.53 ± 0.006 (0.52, 0.54)^d^	0.89 ± 0.008 (0.88, 0.91)	0.37 ± 0.015	0.34, 0.39	1008.25, < 0.000001	Segmented polynomial
S	0.008 ± 0.0005 (0.007, 0.009)	0.002 ± 0.0005 (0.001, 0.003)	−0.006 ± 0.0010	−0.008, −0.004		
Tp	25.1 ± 0.15 (24.8, 25.4)	23.3 ± 0.17 (23.0, 23.6)	−1.8 ± 0.33	−2.44, −1.15		
tip	4.6 ± 0.28 (4.1, 5.2)	6.9 ± 0.31 (6.3, 7.5)	2.2 ± 0.60	1.0, 3.4		
a	0.85 ± 0.035 (0.78, 0.92)	0.96 ± 0.022 (0.92, 1.00)	0.11 ± 0.050	0.01, 0.21	1009.71, < 0.000001	Yang model
c	1.50 ± 0.11 (1.28, 1.71)	1.00 ± 0.0552 (0.89, 1.10)	−0.49 ± 0.140	−0.77, −0.22		
x	0.018 ± 0.0011 (0.016, 0.021)	0.003 ± 0.0006 (0.002, 0.004)	−0.016 ± 0.0015	−0.019, −0.013		
d	22.8 ± 0.058 (22.8, 23.0)	19.9 ± 0.065 (19.8, 20.1)	−3.0 ± 0.13	−3.2, −2.7		
yp	0.53 ± 0.006 (0.52, 0.54)	0.88 ± 0.009 (0.86 0.89)	0.34 ± 0.010	0.32, 0.36	809.168, < 0.000001	Persistency model
t1	21.4 ± 0.13 (21.2, 21.7)	17.5 ± 0.12 (17.3, 17.8)	−3.9 ± 0.25	−4.4, −3.4		
t2	24.3 ± 0.13 (24.0, 24.6)	22.2 ± 0.13 (21.9, 22.4)	−2.1 ± 0.26	−2.6, −1.6		
Persistancy	0	18.5 ± 1.98 (14.6, 22.3)	18.5 ± 3.47	11.7, 25.3		
b4	−0.008 ± 0.0005 (−0.009, −0.007)	−0.008 ± 0.0025 (−0.013, −0.003)	0.000 ± 0.0043	−0.008 0.008		

a Δpar – difference between parameters of Nick chickens (NKpar) and ones of chickens (LYpar). The Δpar were estimated with the indicated models by reparameterizing NKpar as LYpar + Δpar.

b Approximate 95% confidence intervals of Δpar were used to assess whether parameters varied between the Nick (NC) and Lueyang (LY) groups. A confidence interval of Δpar excluded zero (0) indicated that the parameter had a significant difference between LY and NC (SAS Institute Inc., 2008).

c F and P values were obtained by the sum of squares reduction test that indicated any significant difference between the egg-production curves for LY and NC (SAS Institute Inc., 2008).

d Numbers in parenthesis indicate approximate 95% confidence limits of parameters.

### Distribution of VLDLR variants among NC, LohB and LY chickens

Re-sequencing identified 30 SNPs in the VLDLR (Table S2). Of these SNPs, eight accounted for 87% of all variants and were selected as tag SNPs and finally genotyped in LY (n=381), NC (n=91) and LohB (n=50) chickens. VLDLR variants were almost fixed in NC and LohB, with two main haploytpes accounting for 100% of all NC haplotypes and 92.5% of LohB haplotypes (Table 4). In contrast, high sequence polymorphism was observed in LY chickens, for which 14 haplotypes were found (Table 4).

**Table 4:** VLDLR haplotype frequency distributions in Nick Chick, Lohmann Brown and Lueyang chickens.

Name	Haplotype	Lueyang (n=381)	Nick Chick (n=91)	Lohmann (n=50)
LYhap1	GGGGCGCC	0.283	–	–
LYhap2	GGAACACT	0.228	–	–
LYhap3	GTAGTACT	0.108	–	–
LYhap4	GTAATACT	0.089	–	–
**LYhap5**	**ATAATAAT** a	0.079	–	–
LYhap6	GGGACGCC	0.042	–	–
LYhap7	GGGGCACT	0.024	–	–
**LYhap8**	**ATAATACT**	0.032	–	–
LYhap9	GGGGCGCT	0.018	–	–
LYhap10	GGGACGCT	0.021	–	–
LYhap11	GTAGTAAT	0.018	–	–
LYhap12	GTAGTACC	0.018	–	–
LYhap13	GGAGCGCC	0.018	–	–
LYhap14	GGAACGCC	0.021	–	–
NChap1	ATAATAAC	–	0.66	–
NChap2	ATAACAAC	–	0.34	0.925
Lohhap1	ATAACACC	–	–	0.05
Lohhap2	ATGACACC	–	–	0.025

a The haplotypes in bold letters were the two LY haplotypes that showed the highest similarity to two NC haplotypes.

### Comparison of egg production among different VLDLR haplotypes

Of 14 haplotypes found in LY, two (ATAATAAT, ATAATACT) showed high similarity to two NC haplotypes (ATAATAAC, ATAACAAC) and are referred to below as NC-like haplotypes. Egg production was compared between the two NC-like haplotypes and the other LY haplotypes. After discarding haplotypes with frequencies < 5% and combining highly similar haplotypes, we classified the egg production records from 312 LY into four haplotype groups, and then the 13 parameters indicated above were estimated for each group (Table 5). A single significant difference was observed in the peak production rate (Table 5, Figure 2). The differences (Δpar) observed in the remaining parameters were not significant since the 95% confidence interval of Δpar contained zero (0) (Table 5).

**Table 5:** Comparison of egg production parameters between Lueyang chickens with and without NC-like haplotypes.

Haplotypes	ATAATA(A/C)T^a^	GGAACACT	Δpar (95% CI)^b^	GGGGCGCC	Δpar (95% CI)	GTA(A/G)TACT^a^	Δpar (95% CI)	F value	Model
N	42	87		108		75		P value^c^	
Record N	978	2053		2423		1695			
**p**	0.61 ± 0.016	0.55 ± 0.012	−0.06 ± 0.021	0.51 ± 0.011	−0.10 ± 0.020	0.48 ± 0.013	−0.13 ± 0.022	7.16525	Segmented polynomial
	(0.59, 0.65)	(0.53, 0.57)	**(−0.10, −0.00)** d	(0.49, 0.53)	**(−0.14, −0.06)**	(0.46, 0.51)	**(−0.17, −0.09)**	< 0.000001	
s	0.009 ± 0.0013	0.008 ± 0.0010	−0.001 ± 0.0020	0.008 ± 0.0010	−0.001 ± 0.0017	0.006 ± 0.0011	−0.003 ± 0.0020		
	(0.006, 0.011)	(0.006, 0.010)	(−0.004, 0.003)	(0.006, 0.010)	(−0.004, 0.002)	(0.004, 0.008)	(−0.006, 0.000)		
Tp	25.1 ± 0.38	25.1 ± 0.30	0.0 ± 0.50	25.2 ± 0.30	0.1 ± 0.50	25.0 ± 0.38	−0.1 ± 0.55		
	(24.4, 25.8)	(24.5, 25.7)	(−1.0, 1.0)	(24.7, 25.8)	(−0.86, 1.10)	(24.3, 25.8)	(−1.2, 1.0)		
tip	4.9 ± 0.69	4.3 ± 0.55	−0.6 ± 0.92	4.8 ± 0.54	−0.1 ± 0.91	4.9 ± 0.70	−0.0 ± 1.01		
	(3.6, 6.3)	(3.2, 5.4)	(−2.4, 1.2)	(3.7, 5.9)	(−1.9, 1.7)	(3.5, 6.3)	(−2.0, 1.9)		
a	1.00 ± 0.105	0.88 ± 0.071	−0.11 ± 0.13	0.83 ± 0.067	−0.17 ± 0.12	0.75 ± 0.074	−0.25 ± 0.128	7.12442	Yang
	(0.79, 1.20)	(0.74, 1.02)	(−0.36, 0.13)	(0.69, 0.96)	(−0.415, 0.072)	(0.60, 0.89)	(−0.50, 0.003)	< 0.000001	
c	1.29 ± 0.223	1.59 ± 0.238	0.30 ± 0.327	1.46 ± 0.201	0.17 ± 0.301	1.34 ± 0.223	0.045 ± 0.316		
	(0.85, 1.73)	(1.12, 2.06)	(−0.34, 0.94)	(1.06, 1.85)	(−0.42, 0.76)	(0.90, 1.78)	(−0.57, 0.67)		
x	0.019 ± 0.003	0.018 ± 0.0023	−0.000 ± 0.0038	0.018 ± 0.002	−0.000 ± 0.0038	0.017 ± 0.0029	−0.002 ± 0.0042		
	(0.013, 0.024)	(0.014, 0.023)	(−0.008, 0.007)	(0.014, 0.023)	(−0.008, 0.007)	(0.011, 0.022)	(−0.010, 0.006)		
d	22.7 ± 0.16	23.0 ± 0.11	0.3 ± 0.20	22.9 ± 0.11	0.1 ± 0.20	22.7 ± 0.15	−0.1 ± 0.22		
	(22.4, 23.1)	(22.8, 23.2)	(−0.1, 0.7)	(22.7, 23.1)	(−0.2, 0.5)	(22.4, 23.0)	(−0.5, 0.4)		
**yp**	0.62 ± 0.018	0.56 ± 0.013	−0.06 ± 0.022	0.52 ± 0.011	−0.1 ± 0.021	0.49 ± 0.014	−0.13 ± 0.023	7.08413	Persistency
(0.58, 0.65)	(0.53, 0.58)	**(−0.11, −0.02)**	(0.50, 0.54)	**(−0.14, −0.06)**	(0.46, 0.51)	**(−0.18, −0.09)**	< 0.000001		
t1	21.0 ± 0.33	21.5 ± 0.25	0.5 ± 0.42	21.4 ± 0.24	0.4 ± 0.41	20.9 ± 0.31	0.2 ± 0.52		
	(20.4, 21.7)	(1.1, 2.0)	(−0.3, 1.3)	(0.9, 1.9)	(−0.4, 1.2)	(20.3, 21.5)	(−0.8, 1.2)		
t2	24.3 ± 0.34	24.4 ± 0.26	0.1 ± 0.43	24.3 ± 0.25	0.0 ± 0.42	24.3 ± 0.33	0.1 ± 0.53		
	(23.6, 25.0)	(23.9, 24.9)	(−0.7, 1.0)	(23.8, 24.8)	(−0.8, 0.9)	(23.6, 24.9)	(−0.9, 1.2)		
Persistency	0	0	–	0	–	0	–		
b4	−0.009 ± 0.0014	−0.008 ± 0.0010	0.001 ± 0.0017	−0.008 ± 0.0009	0.001 ± 0.0017	−0.006 ± 0.0011	−0.002 ± 0.0021		
	(−0.012, −0.006)	(−0.010, −0.006)	(−0.003, 0.004)	(−0.010, −0.006)	(−0.002, 0.004)	(−0.008, 0.004)	(−0.006, 0.002)		

a ATAATA(A/C)T were defined as NC-like haplotypes based on their high similarity to the NC haplotypes ATAA(T/C)AAC.

b Δpar represents difference between parameters of the three haplotype groups (Parcont) and ones of the ATAATA(A/C)T group (Parcase), and was estimated using the models indicated in the Table by reparameterizing Parcont as Parcase + Δpar. The numbers in parentheses indicate the approximate 95% confidence interval (CI) of parameters. The CIs of Δpar that exclude zero (0) indicate that the parameters have significant between two haplotype groups (SAS Institute Inc., 2008).

c F and P values were determined by the sum of squares reduction test which indicated whether there was a significant difference in the egg-production curves fitted for the different haplotype groups (SAS Institute Inc., 2008).

d Numbers in bold indicate the 95% CI of Δpar that excluded zero (0), indicating that the corresponding parameters were significantly different between ATAATA(A/C)T and the other haplotype groups.

### Haplotype-specific expression analysis of VLDLR

**Figure 3:**
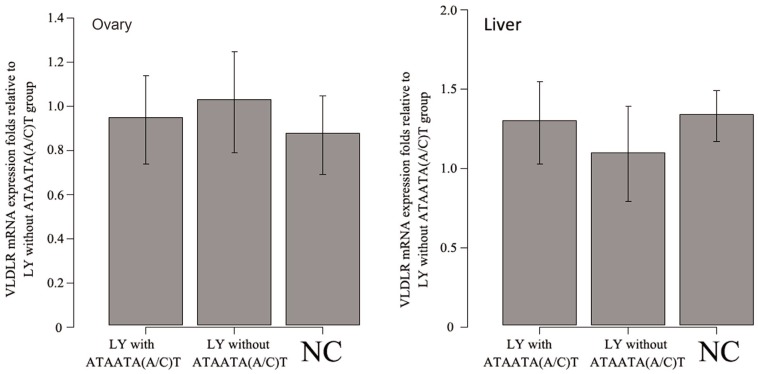
Comparison of VLDLR mRNA expression in Lueyang (LY) chickens with or without ATAATA(A/C)T and in Nick Chick (NC) chickens. LY without the ATAATA(A/C)T group was used as a calibrator. The expression levels of VLDLR mRNA in LY with ATAATA(A/C)T and NC are shown as the fold-change relative to the calibrator. The columns represent the mean ± SD of the fold change for LY with ATAATA(A/C)T (n=6), LY without ATAATA(A/C)T (n=6) and NC (n=6) in liver and ovary. There was no significant difference (p > 0.05) in VLDLR mRNA expression among the groups within a given organ (liver and ovary). Statistical significance was test by ANOVA followed by Duncan's multiple range test.

The level of VLDLR mRNA expression was compared among the LY and NC-like haplotypes (n=6), LY without the haplotypes (n=6) and NC (n=6). There was no significant difference in the expression of VLDLR mRNA in ovary and liver between any two groups (Figure 3).

### Detection of the RO mutation in LY chickens

**Figure 4:**
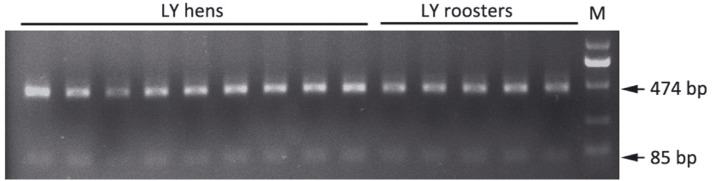
Detection of the RO mutation in LY hens (n=50) and roosters (n=30). The RO mutation was detected using PCR-RFLP. Wild-type individuals had two bands (85 bp and 474 bp) and those with the RO mutation had four bands (85 bp, 108 bp, 451 bp and 474 bp).

The RO mutation is a missense mutation that leads to a failure to lay eggs and results in hyperlipidemia (Bujo *et al.*, 1995). Although there is no change in reproductive function, roosters with this mutation transmit it to one-half of their daughters (Elkin and Zhong, 2002). To examine the distribution of the RO mutation in LY and assess whether it is a major factor resulting in the low egg production of LY, 50 LY hens and 30 cocks were screened for the mutation. All individuals showed two bands (85 bp and 474 bp), indicating that the mutation did not exist in the samples that were tested (Figure 4).

## Discussion

We have previously observed two important characteristics of LY chickens, namely, their low egg production and large intraspecies variability. In this study, all birds from the same hatch were reared in the same environment. This experimental design allowed us to conclude that these features were mainly determined by genetic factors. Large intraspecies variability implies that some variants that regulate egg production may be separable in the LY breed, which, thus, could provide an interesting animal model for identification of the relevant mutations.

Although VLDLR is a promising candidate gene for modulating egg production. our knowledge of the relationship between VLDLR variants and egg production is still incomplete; the only mutation known to adversely affect hen reproduction is the RO mutation (Bujo *et al.*, 1995). No studies have specifically sought to map the distribution of VLDLR variants among different breeds such that we do not know whether there are some favorable, specifically-selected variants in the VLDLR. In this study, the genome sequence covering the whole VLDLR and a 5 kb region upstream was re-sequenced and eight SNPs accounting for 87% of all variants were genotyped in two commercial egg-laying breeds (NC and LohB) and LY. The data obtained from this sequencing showed that: (1) VLDLR variants were almost fixed in the commercial breeds, whereas there was considerable haplotype diversity in LY (Table 4) and (2) two LY haplotypes, similar to NC haplotypes, were significantly associated with high egg production (Table 5).

A decrease in genetic variation can be caused by selection, inbreeding and genetic drift. Clarification of the roles of these factors in shaping the distribution patterns of VLDLR variants among these breeds is key to understanding the influence of VLDLR variants on egg production. For breeders, inbreeding is a useful way of fixing traits within a short period of time and has been widely used to breed pure lines. However, the effect of inbreeding on genome variants is general instead of being confined to a specific gene (Charlesworth, 2003). Genetic drift can also change the allele frequency, leading to the fixation or disappearance of variants through random sampling (Masel, 2011). As shown here, four haplotypes were found in NC and LohB (Table 4). These haplotypes showed high similarity between the two breeds, with the first four bases being completely conserved and NChap2 occurring in LohB at a particularly high frequency of 0.925 (Table 4). NC is a popular white-egg laying breed, initially bred by H&N International based on the Kimber Leghorn in 1945. LohB was bred from New Hampshire and other breeds that lay brown eggs. The different genetic backgrounds of these breeds suggests that the possibility of highly similar haplotypic profiles appearing in different breeds should be small if inbreeding and genetic drift are the determining factors.

The effect of selection on genetic variants within the genome is immense. Human-driven selection not only increases the frequency of favorable variants in the population, but also leads to a reduction or loss of nucleotide diversity at some linked neutral loci, a phenomenon often referred to as "genetic hitchhiking" or "selective sweep" (Elferink *et al.*, 2012). Sundström *et al.* (2004) detected Z chromosome variability in 13 introns of nine genes, including VLDLR, and confirmed that there was a signature of selection on chromosome Z. If the decrease in haplotype diversity in NC and LohB were caused by selection, we would expect these haplotypes to represent functional haplotypes associated with high egg production. Fortunately, considerable haplotypic diversity was maintained in LY, which provides an opportunity to analyze the phenotypic effect of these haplotypes. As shown here, two NC-like haplotypes were significantly associated with high peak production (Table 5, Figure 2), a finding compatible with "selective sweep" having a key role in reducing VLDLR variations.

Han *et al.* (2009) suggested that the level of VLDLR mRNA expression in ovary was a determining factor in the reproductive phenotype. However, our results showed that the expression level of this receptor was not higher in the NC or NC-like haplotypes than in the other haplotypes (Figure 3), which indicated that high egg production was not necessarily associated with enhanced VLDLR expression. On the other hand, variations in mRNA expression do not necessarily couple with changes in protein activity and function (Nikinmaa and Waser, 2007). After being synthesized, VLDLR needs to be transferred to the oocyte periphery to function (Bujo *et al.*, 1994). Fluorescence *in situ* hybridization and ligand-binding assays will be needed to clarify whether the two phenotype-associated haplotypes are correlated with changes at the protein level.

In summary, our data indicate that VLDLR variants were almost fixed in selectively bred NC and LohB chickens, whereas haplotypic diversity was maintained in LY chickens. The finding that different egg-laying breeds shared similar haplotypic profiles and that the haplotypes exerted a phenotype effect suggested that selection may be a key factor in shaping the current distribution patterns of VLDLR variants. To our knowledge, this is the first report to specifically focus on the distribution of VLDLR variants in commercial and indigenous breeds of chicken. The results provide new support for VLDLR as a promising candidate gene for modulating egg production.

## Supplementary material

The following online material is available for this article:

Table S1 - Primer sets used for PCR and DNA sequencing of the VLDLR gene.

Table S2 - Information for all SNPs found in the VLDLR
