# Supplementary material for: The association of very low-density lipoprotein receptor (VLDLR) haplotypes with egg production indicates VLDLR is a candidate gene for modulating egg production
Source: Genet Mol Biol. 2016 Jul 14;39(3):380–91. doi: 10.1590/1678-4685-GMB-2015-0206 (PMC5004830; doi:10.1590/1678-4685-GMB-2015-0206)
Supplement: Supplementary file 1 [file 1415-4757-gmb-1678-4685-GMB-2015-0206-Suppl01.pdf]

Table S1 - Primer sets used for PCR and DNA sequencing of the VLDLR gene.

Primer ID	Primer sequence (5'-3')	Product size (bp)
V_1F	CTGTCCCACAATGCTCTA	1420
V_1R	AGTGACCCTGGTAAATGG	
V_3F	TGCCGATGTAGATGTGGT	1354
V_3R	TCCTTCTGTTAGCCTGTGA	
V_4F	AAAGCCTGGATAAGAGCG	771
V_4R	CTACTGCAATGGTGGTGC	
V_5F	GCGGTGGTATGCTGTAGG	1572
V_5R	AGGCAAGAAGGCAAATGG	
V_6F	TGCTGAACTGTGCCGTAA	881
V_6R	AGCCACCATTTCATCCCT	
V_7F	GTCCTGGAAATGTGATGG	1040
V_7R	GGTAGCCAGATGGTCAGC	
V_8F	CATCTGGCTACCAATACC	912
V_8R	GCAGTTCCCATCTTCACA	
V_9F	TAACTTGGTCCTTCTTCTA	993
V_9R	CTTATTTGATACTTGCCTC	
V_10F	GCCAAATCTGTATCAACC	1007
V_10R	ACGGGTATCAATAGAGGC	
V_11F	ACCCGTGATAAAGTTGGA	827
V_11R	AAGACTGGCAGAGTTTCC	

V_12F	AAGTGGTAACCTTCCCTG	1142
V_12R	TTGCCTCTTACAAGTTTAGT	
V_13F	AGAGGCAAACATGACAGT	1278
V_13R	TAAGACATTAAGCCCACA	
V_14F	TGCCAAGAGTGCTCAAAG	1129
V_14R	TCCAAGAATGGAGGAAGT	
V_15F	AGTGAAGACTTGCGTTGT	1037
V_15R	CTGCTTTGATACCCATCT	
