# Supplementary material for: The association of very low-density lipoprotein receptor (VLDLR) haplotypes with egg production indicates VLDLR is a candidate gene for modulating egg production
Source: Genet Mol Biol. 2016 Jul 14;39(3):380–91. doi: 10.1590/1678-4685-GMB-2015-0206 (PMC5004830; doi:10.1590/1678-4685-GMB-2015-0206)
Supplement: Supplementary file 2 [file 1415-4757-gmb-1678-4685-GMB-2015-0206-Suppl02.pdf]

Table S2 - Information for all SNPs found in the VLDLR gene.

SNP ID	Description[a]	Position in VLDLR gene	dbSNP accession no.
SNP3	g.26417373 T>C	intron1	rs16775985
SNP4	g.26417380 C>T	intron1	rs16775986
SNP5	g.26417409 G>T	intron1	new
SNP6	g.26417609 C>G	intron1	new
**SNP8[b]**	**g.26419086 G>A**	**intron1**	**rs312633123**
**SNP9**	**g.26420908T>G**	**intron2**	**new**
SNP13	g.26422763T>C	intron4	new
**SNP14**	**g.26423124A>G**	**intron4**	**new**
**SNP19**	**g.26424657G>A**	**exon6,synonymy**	**rs317219970**
SNP20	g.26424907T>C	intron7	rs312397794
SNP21	g.26425237G>A	intron7	new
SNP24	g.26425727A>G	intron9	rs14777628
SNP25	g.26425926T>A	intron9	new
SNP26	g.26426241T>C	intron10	rs314956051
SNP27	g.26426244G>A	intron10	rs317006300
SNP28	g.26426267T>C	intron10	rs317616384
SNP29	g.26426272C>T	intron10	rs315755231
SNP30	g.26426369C>T	intron10	rs316405899
SNP31	g.26426376C>T	intron10	rs315181270
**SNP32**	**g.26426398T>C**	**intron10**	**rs317314828**
SNP35	g.26426760CT>AG	intron11	new
SNP36	g.26426908T>C	intron11	rs316956892
SNP39	g.26427704C>T	exon14, synonymy	rs317592904

**SNP41**	**g.26427922A>G**	**intron14**	**rs314567990**
**SNP46**	**g.26429604A>C**	**intron15**	**rs14777635**
SNP48	g.26429706A>C	exon16, synomymy	rs14777636
SNP49	g.26430448G>A	intron17	rs14777642
SNP52	g.26431178G>A	3' flanking region	rs318038559
**SNP53**	**g.26431186C>T**	**3' flanking region**	**rs314284862**
SNP54	g.26431361G>A	3' flanking region	new

[a]Genomic positions of SNPs are based on the chicken genome galGal4 assembly (http://genome.ucsc.edu).

[b]SNPs shown in bold letters were used in the haplotype association study.
